# Effect of Mg on the Microstructure and Corrosion Resistance of the Continuously Hot-Dip Galvanizing Zn-Mg Coating

**DOI:** 10.3390/ma10080980

**Published:** 2017-08-22

**Authors:** Anping Dong, Baoping Li, Yanling Lu, Guoliang Zhu, Hui Xing, Da Shu, Baode Sun, Jun Wang

**Affiliations:** 1 School of Materials Science and Engineering, Shanghai Jiao Tong University, Shanghai 200240, China; apdong@sjtu.edu.cn (A.D.); xinghui@sjtu.edu.cn (H.X.); dshu@sjtu.edu.cn (D.S.); bdsun@sjtu.edu.cn (B.S.); junwang@sjtu.edu.cn (J.W.); 2 Shanghai Key Lab of Advanced High-temperature Materials and Precision Forming, Shanghai 200240, China; 3 Hechi Industry and Information Committee, Hechi 547000, China; 4 Department of Nuclear Materials Science and Engineering, Shanghai Institute of Applied Physics, Chinese Academy of Sciences, Shanghai 201800, China; 5 State Laboratory of Metal Matrix Composites, Shanghai Jiao Tong University, Shanghai 200240, China

**Keywords:** continuously hot-dip galvanizing, Zn-Mg coating, TEM, corrosion resistance

## Abstract

The microstructure of continuously hot-dip galvanizing Zn-Mg coating was investigated in order to obtain the mechanism of the effects of Mg on the corrosion resistance. In this paper, the vertical section of the Zn-0.20 wt % Al-Mg ternary phase diagram near the Al-low corner was calculated. The results indicates that the phase composition of the Zn-0.20 wt % Al-Mg ternary phase diagram near the Al-low corner is the same as Zn-Mg binary phase diagram, suggesting Al in the Zn-Mg (ZM) coatings mainly concentrates on the interfacial layer between the coating and steel substrate. The microstructure of continuously hot-dip galvanizing ZM coatings with 0.20 wt % Al containing 1.0–3.0 wt % Mg was investigated using tunneling electron microscopy (TEM). The morphology of Zn in the coating changes from bulk to strip and finally to mesh-like, and the MgZn_2_ changes from rod-like to mesh-like with the Mg content increasing. Al in the ZM coatings mainly segregates at the Fe_2_Al_5_ inhibition layer and the Mg added to the Zn bath makes this inhibition layer thinner and uneven. Compared to GI coating, the time of the first red rust appears increases by more than two-fold and expansion rate of red rust reduces by more than four-fold in terms of salt spray experiment. The ZM coating containing 2.0 wt % Mg has the best corrosion resistance. The enhanced corrosion resistance of ZM coatings mainly depends on different corrosion products.

## 1. Introduction

In recent years, hot-dip galvanizing Zn coatings containing Mg, referred to as a promising next generation of Zn-based coatings, have received considerable attention, primarily due to their superior corrosion resistance with respect to GI (Zn-0.20 wt % Al) coating [1,2,3,4,5,6,7] or Zn-Al coatings [8,9].

The Zn-based coatings containing Mg can be roughly classified into two types: Zn-Al-Mg coatings with high Al and Zn-Al-Mg coatings with very low Al or without Al. For Zn-Al-Mg coatings with high Al, the role of Al is to impede the oxidation of Zn liquid and enhance corrosion resistance. The conventional continuously hot-dip galvanizing technique was applied to mass-produce this type of Zn based coatings [3,4,5,8,9,10,11,12]. For Zn-Al-Mg coatings with very low Al or without Al, hereafter referred to as Zn-Mg coatings, other techniques, such as physical vapor deposition (PVD) [7,13,14], batching galvanizing [6,15], electrodeposition (EG) [16,17,18], and the two-step method [2], were selected to produce these Zn-based coatings to avoid interference of the oxidation of Mg with production. For PVD and the two-step method, the oxidation of Mg can be eliminated completely. For batching galvanizing method, prior to hot-dip galvanizing, the oxidation layer of Mg can be sweep away from the surface of Zn liquid manually. With the Al in the Zn coating increasing, the welding [19] and cathodic protection performance [20] will deteriorate. In this regard, the Zn-Mg coatings with a low Al content is beneficial to expand application in different fields. Al (0.20 wt %) was added into the Zn bath in order to utilize the processing parameters of the GI coating. Prosek investigated the corrosion resistance of Zn-Mg alloys and reported that when the Mg content in the Zn-Mg alloys was less than 8.0 wt %, the corrosion resistance of Zn-Mg alloy increased with the Mg content increasing after exposure to humid air [3]. To date, the systematical investigation relevant to microstructure and corrosion resistance of the continuously hot-dip galvanizing Zn-Mg coatings with relatively high Mg contents is scarce.

Most of the studies focused on high Mg and Al content addition to the zinc coating. However, it is not good for producing high-quality hot-dip galvanized automobile sheet, mainly because high Al content leads to excessive zinc dross, thus affecting the surface quality of the hot-dip galvanized automobile sheet, and increase zinc consumption. In addition, too high Mg results in severe oxidation in the galvanizing temperature. In this study, we propose the low Mg and Al content in the zinc coating to avoid the oxidation of zinc liquid and improve wettability. The Mg in hot-dip Zn coatings can inhibit the grain boundary corrosion and improve the adherence of the corrosion product layer on the coating, thereby ameliorating the corrosion resistance.

In this paper, the Zn-0.20 wt % Al-Mg (ZM)-coated steel sheets with 1.0–3.0 wt % Mg were prepared through the continuously hot-dip galvanizing with the same processing parameters as GI coated steel sheets in the laboratory. The vertical section of the ternary Zn-0.20 wt % Al-Mg phase diagram near the Al-low corner was calculated using the thermodynamic data of the available literature [21] to predict the phase composition. The microstructure and corrosion behaviors of ZM coatings were investigated in our previous research [22]. In this part, we further investigate the microstructure of ZM coatings using tunneling electron microscopy (TEM). In addition, corrosion resistance of ZM coatings is also studied to optimize the coating composition.

## 2. Experimental

### 2.1. Phase Diagram Calculation

To predict the phase composition of ZM coating, the vertical section of this ternary phase diagram with 0.20 wt % Al near Al-low corner at 460 °C (typical hot-dip galvanizing temperature) was calculated using the ThermoCalc program (CALPHAD, Thermo-Calc , Stockholm, Sweden) in terms of the literature [21].

### 2.2. Materials and Microstructure Characterization

Three sets of continuously hot-dip galvanizing ZM-coated steel sheets and GI-coated steel sheets were investigated in this paper by using the continuously hot-dip galvanizing simulator in the Baosteel Group Corporation. The temperature of the steel sheet entering the zinc pot is about 780 °C, the hot-dip galvanizing temperature is 460 °C, and the immersion time is 3 s. The steel substrate is the IF steel sheet with a thickness of 0.5 mm and its chemical composition is listed in Table 1.

Nominal compositions (wt %) of GI and ZM coatings are Zn-0.20 Al, Zn-0.20 Al-1.0 Mg, Zn-0.2 0Al-2.0 Mg, and Zn-0.2 Al-3.0 Mg, hereafter referred to as GI, ZM1, ZM2, and ZM3, respectively.

The microstructure of ZM coatings and the interfacial layer between the coating and steel substrate were investigated using TEM (JEM-2000EX, JEOL, Tokyo, Japan. Accelerating voltage: 160 KV). The TEM samples less than 100 nm in thickness were prepared with a focused ion beam (FIB) system (FEI Dual Beam 820, FEI, Hillsboro, OR, USA).

### 2.3. Evaluation of Corrosion Resistance

A salt spray test and linear polarization method were conducted to evaluate the corrosion resistance of GI- and ZM-coated steel sheets. At least three identical samples were used for every chemical composition to obtain reliable data.

For the salt spray test, the samples of 40 mm × 40 mm were cut from the perfect areas of GI- and ZM- coated steel sheets. Prior to the test, the surface contaminants on samples were removed by an ultrasonic treatment in acetone solution, and then the periphery of samples was sealed with epoxy resin. Finally, the samples were introduced to a standard salt spray chamber (FQY, Yuanyao, Dongguan, China) according to DIN EN ISO 9227, detailed parameters of which are shown elsewhere [1].

For the linear polarization experiment, samples of 10 mm × 10 mm were cut from the perfect areas of GI- and ZM-coated steel sheets. The samples for linear polarization experiment were prepared as follows: first, the samples were cleaned as in the case of the salt spray test. Secondly, one surface of samples was welded with a Cu wire and then this surface of samples was sealed together with the periphery of samples using epoxy resin. A standard three-electrode system (CHI 660 C, CHI, Wildwood, NJ, USA) was used with platinum as the counter electrode, SCE (saturated calomel electrode) as the reference electrode, and samples as the working electrodes. The samples were immersed in an aerated and quiescent 5 wt % NaCl solution for 15 min to stabilize their open potentials before the linear polarization experiment. After that, linear polarization measurements were carried out using a sweep rate of 0.01 V/s and a potential range from −1.8 to 0.2 V.

### 2.4. Characterization of Corrosion Products

The phase composition of corrosion products on GI and ZM coatings after the salt spray test were identified using XRD (Philips X’pert, PANalytical B.V., Almelo, The Netherlands) and Fourier-transformed infrared spectroscopy. For the XRD experiment, the scanning range of 2*θ* is from 10° to 70°. The scanning rate is 0.02°/s. For the Fourier-transformed infrared spectroscopy experiment, 0.70 mg of corrosion products were mixed with a spectroscopic-grade potassium bromide (KBr) powder to obtain 140 mg of a mixture. The mixture was ground in a mortar and pressed into pellets in a die. Spectra were collected by adding 16 scans at 8 cm^−1^ resolution in the range from 400 to 4000 cm^−1^. A background spectrum was obtained from a pure KBr pellet of the same weight and size. The corrosion products on GI and ZM coatings were also investigated using SEM (JEOL, Tokyo, Japan).

## 3. Results and Discussion

### 3.1. Zn-Al-Mg Ternary Phase Diagram

**Figure 1:**
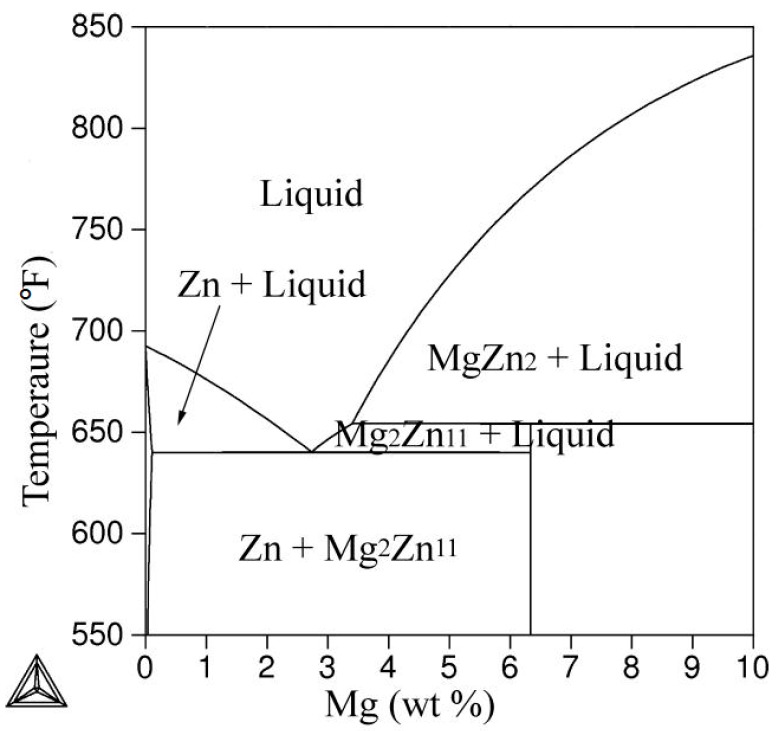
Vertical section of Zn-0.2 wt % Al-Mg ternary phase diagram near the Al-low corner.

Figure 1 shows the vertical cross-section of the Zn-0.20 wt % Al-Mg ternary phase diagram near the Al-low corner. Compared with the Zn-Mg binary phase diagram [23], it is found that the phase composition of this ternary phase diagram is the same as in the Zn-Mg binary phase diagram near the Al-low corner, namely Zn plus Mg_2_Zn_11_, implying no Mg-containing intermetallic compounds form in the coating during the equilibrium solidification. In this regard, the Zn-0.20 wt % Al-Mg coating can be considered as Zn-Mg coating. The temperature of eutectic point of Zn-0.20 wt % Al-Mg ternary system is about 637 K, which is very close to that of the Zn-Mg binary system. Additionally, it is of interest to note that no Al-containing intermetallic compounds form in the coating by means of this phase diagram, suggesting Al added to the Zn bath may be still segregate at the interfacial layer between the coating and steel substrate.

### 3.2. Microstructure

**Figure 2:**
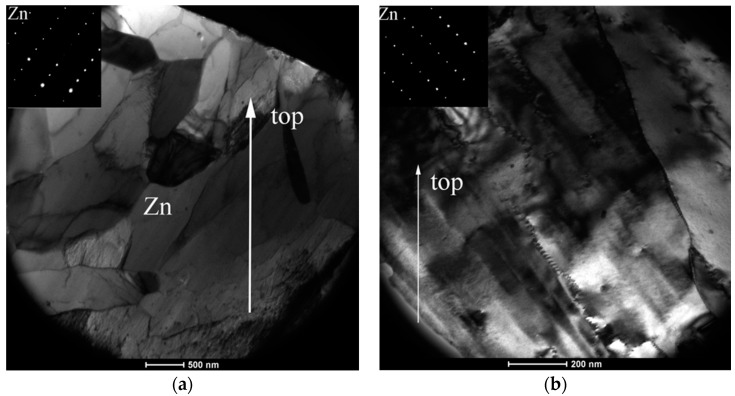

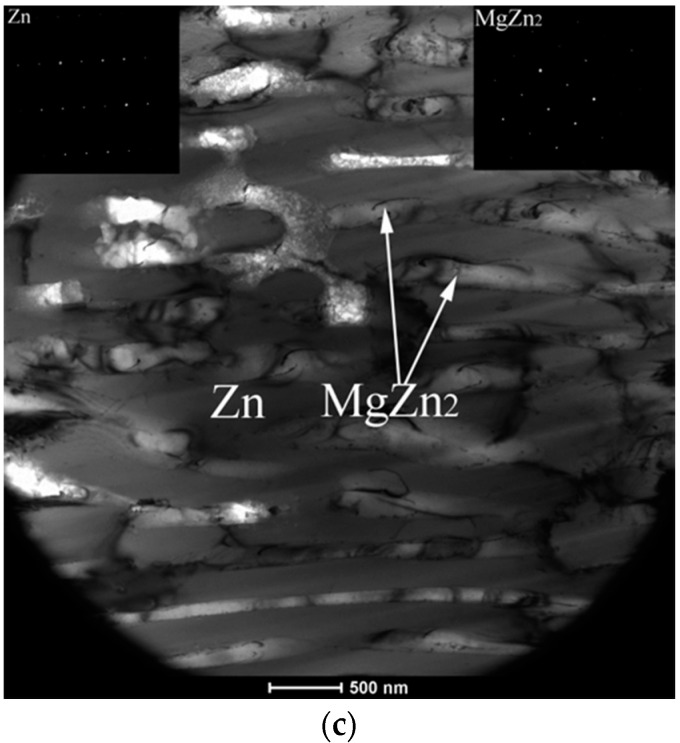
TEM micrographs of Zn on ZM coatings, (**a**) ZM1; (**b**) ZM2; and (**c**) ZM3.

**Figure 3:**
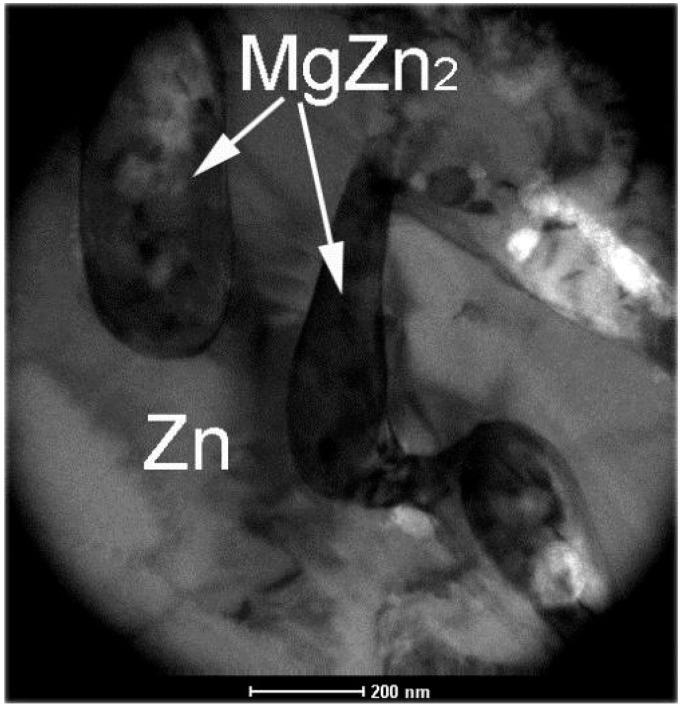
The TEM micrograph of rod-like MgZn_2_.

The microstructure of ZM coatings was investigated using SEM in our previous published paper [22]. In this paper, the TEM observation was performed to investigate further the coating microstructure and the interfacial layer between the coating and steel substrate. Figure 2 shows the TEM micrographs of Zn in ZM coatings. As seen in the Figure 2, the Zn morphology changes with different Mg into the Zn bath. Many bulk grains are observed in ZM1 coating (Figure 2a). With the Mg increasing to 2.0 wt %, many long strip Zn grains are observed in ZM2 coating (Figure 2b). With Mg increasing to 3.0 wt %, the mesh-like Zn grains are observed as shown in ZM3 coating (Figure 2c). Similarly, the morphology of MgZn_2_ changes with the Mg content in the ZM coating. The rod-like MgZn_2_ is observed in ZM2 coating (Figure 3). The mesh-like MgZn_2_ is observed in ZM3 coating (Figure 2c).

**Figure 4:**
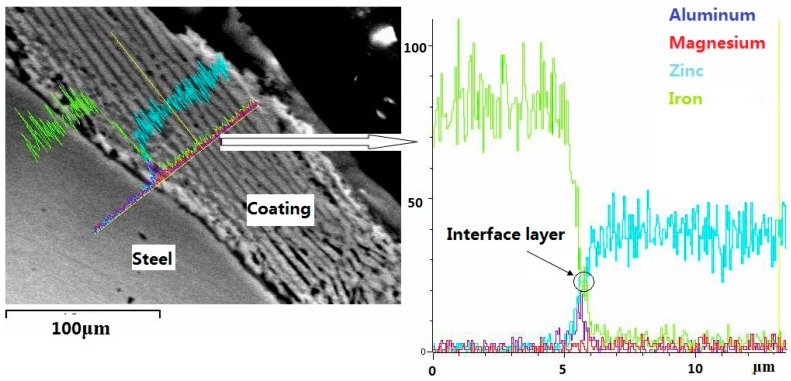
Linear scanning micrographs of the ZM3 coating.

In general, an Al-rich inhibition layer (Fe_2_Al_5_) with a thickness of 500 nm was formed between the steel substrate and the coating for the GI coating which was produced by the continuously hot-dip galvanizing method [24]. To find out whether there is still an Al-rich inhibition layer between the coating and steel substrate for ZM coatings, a representative linear scan is shown in Figure 4. As seen in Figure 4, an abrupt Al-rich peak is obviously observed, implying that there exists an Al-rich inhibition layer.

**Figure 5:**
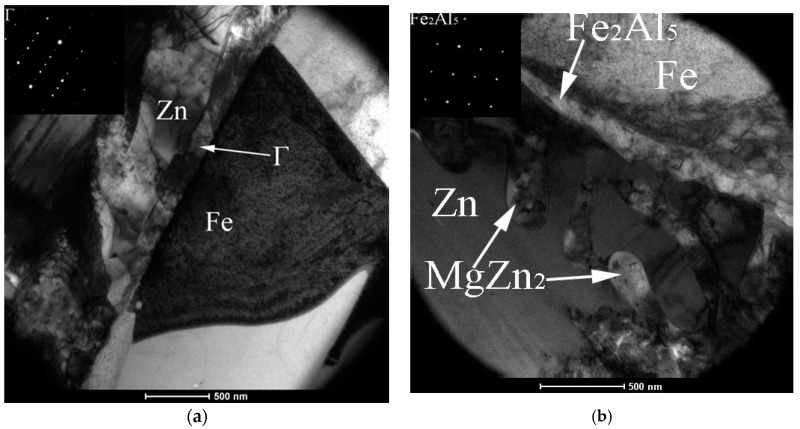

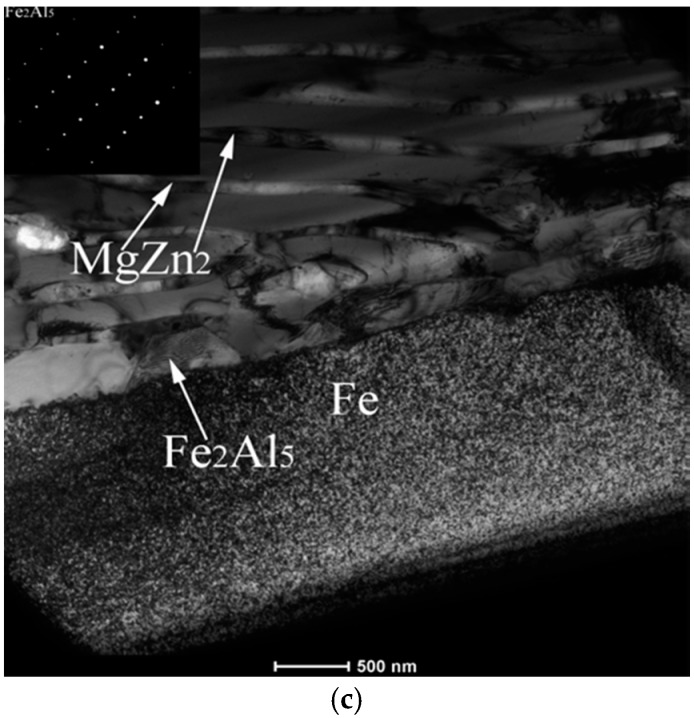
The TEM microgpraphs of the interfacial layer on the ZM coatings, (**a**) ZM1; (**b**) ZM2; and (**c**) ZM3.

Figure 5 shows the TEM micrographs of the interfacial layer on ZM coatings, and a Γ phase is found on the interfacial situation of the ZM1 coating (Figure 5a). However, for ZM2 and ZM3 coatings, there exists a Fe_2_Al_5_ interfacial layer (Figure 5b,c). For these ZM coatings, the thickness of the interfacial layer is about 300 nm which is obviously thinner than that formed in the GI coating [24]. Additionally, it is found that the Fe_2_Al_5_ inhibition layer in some regions is very thin, as seen in the ZM3 coating. This result implies that the steel sheet surface may not be covered by the Fe_2_Al_5_ inhibition layer, resulting in the formation of the Fe-Zn compound in local regions of the Zn coating. As a result, adherence of the Zn coating to the steel substrate decreases due to the formation of the Fe-Zn compound [25,26]. It can be inferred that Mg can affect the formation of the Fe_2_Al_5_ inhibition layer. To fully inhibit the formation of Fe-Zn compounds, Al added to the Zn bath containing Mg should increase accordingly.

### 3.3. Corrosion Resistance

The results of the salt spray experiment are showed in Table 2. The first white rust time for ZM and GI coatings is the same (two days). It is noteworthy that, with respect to GI, a more than two times increase in the first red rust time and a more than three times increase in the severe red rust time are observed for ZM coatings. The best corrosion resistance can be obtained for the ZM2 coating. The time from the first red to severe red rust increases by four times for ZM coatings compared to the GI coating. A similar result was reported in a previous study where the Zn-Mg coating was prepared by electrodeposition and its corrosion resistance evaluated by a salt spray test [16]. From the salt spray experiment results, the corrosion resistance of GI and ZM coatings from good to poor is as follows: ZM2 > ZM1 > ZM3 > GI.

**Figure 6:**
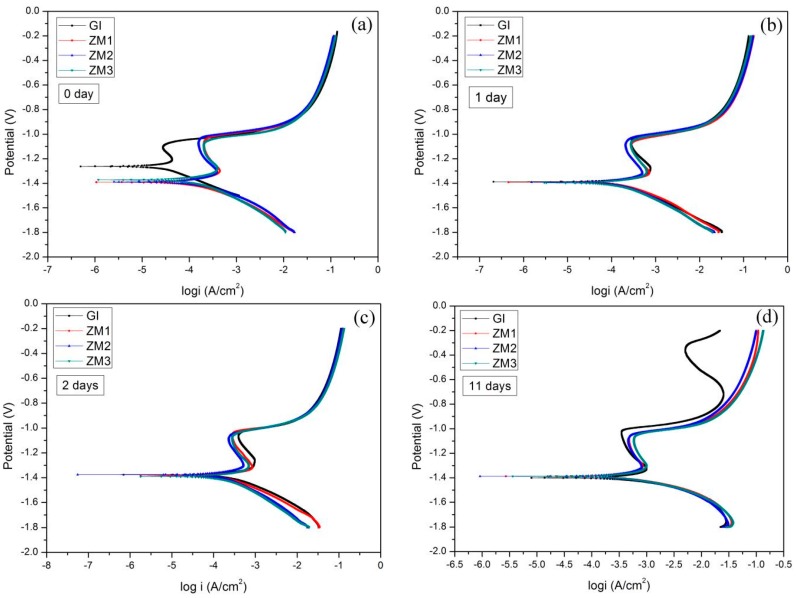
Linear polarization curves of GI and ZM coatings after different immersion times in 5 wt % NaCl solution. (**a**) 0 day; (**b**) 1 day; (**c**) 2 days; (**d**) 11 days.

Figure 6 shows linear polarization curves of GI and ZM coatings after different immersion times in 5.0 wt % NaCl solution. When the immersion time is less than or equal to two days, the anodic and cathodic parts of the linear polarization curves for GI and ZM coatings are similar, especially on the very high potential or rather low potential regions. After 11 days of immersion, on the anodic part of the linear polarization curve for the GI coating, the second anodic electrochemical reaction is observed near −0.7 V, implying corrosion possibly occurred on the steel substrate.

**Figure 7:**
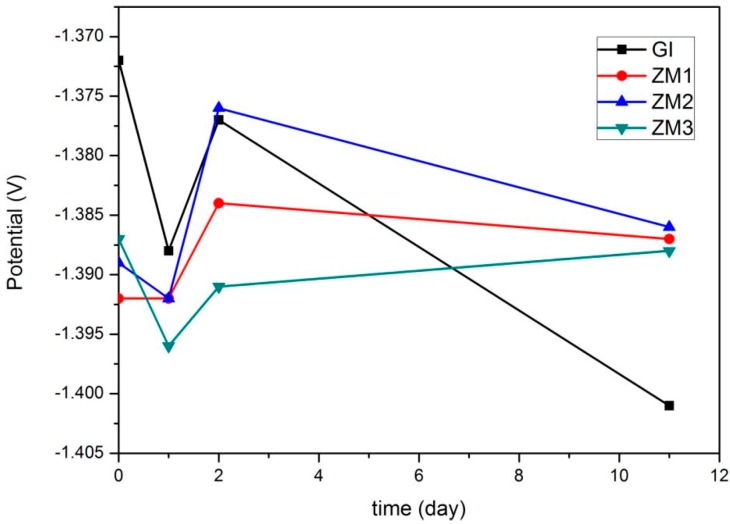
Free corrosion potential curves of GI and ZM coatings versus time after different immersion times in 5 wt % NaCl solution.

**Figure 8:**
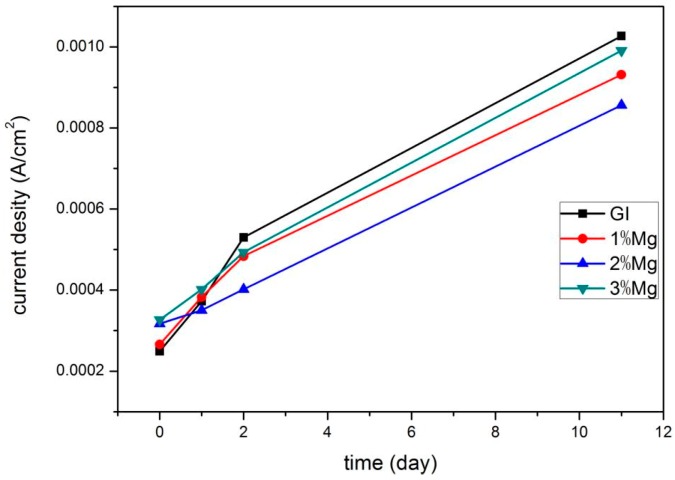
Free corrosion current density curves of GI and ZM coatings versus time after different immersion times in 5 wt % NaCl solution.

Free corrosion potential and free corrosion current density curves versus time, derived from linear polarization curves, are shown in Figure 7 and Figure 8, respectively. These curves clearly characterize the corrosion resistance.

As indicated in Figure 7, it can be seen that the general variation tendency of the free corrosion potential for GI and ZM coatings is the same. At the very initial stage of corrosion, the free corrosion potentials for all samples were relatively high, which may be connected with the formation of a thin semiconductor metal oxide layer (ZnO or a mixture of ZnO and MgO) on the coating surface in contact with atmosphere [3]. However, the free corrosion potentials for ZM coatings are lower than that of the GI coating because magnesium oxide has a lower potential than zinc oxide [3]. After one day of immersion, the free corrosion potentials for all samples reduce due to the active corrosion of the metal coatings. The free corrosion potential for the GI coating is still higher than that of the ZM coatings. After two days of immersion, the free corrosion potentials for all the samples increase due to the formation of corrosion products which can impede the infiltration of aggressive corrosion mediums, such as Cl^−^ ions. It is noteworthy that the free corrosion potential of the ZM2 coating is slightly higher than that of the GI coating, suggesting its corrosion products probably differ with the GI coating. After 11 days of immersion, the free corrosion potentials for the ZM coatings are higher than the GI coating, in which the ZM2 coating possesses the highest free corrosion potential, suggesting the corrosion products formed on ZM coatings have a better protection ability to the steel substrate with respect to the GI coating [27].

As indicated in Figure 8, at the very initial stage of corrosion, it is obvious that the free corrosion current density of the GI coating is lower than that of the ZM coatings, suggesting Mg added to the Zn bath can accelerate the corrosion process. This result was reported in the literature where MgZn_2_ was corroded preferentially in the Zn-Al-Mg coating [1,28]. After one day of immersion, the free corrosion current density of the ZM2 coating is slightly lower than that of the GI coating, however, the rest of the ZM coatings are still higher than that of the GI coating, indicating the corrosion products on the ZM2 coating have the best protection ability. After two days of immersion, the free corrosion current density for all ZM coatings is clearly lower than that of the GI coating. The lowest free corrosion current density is still observed for the ZM2 coating. Based on the results of the linear polarization experiment, corrosion resistance of GI and ZM coatings is as follows: ZM2 > ZM1 > ZM3 > GI. This is in accordance with the result of the salt spray experiment. The corrosion resistance is closely connected with the microstructure of Zn-Mg coatings. When 1.0 wt % Mg was added to the Zn bath, the microstructure of the Zn-Mg coating is similar to the GI coating [22], its corrosion behavior is also similar to GI except for elimination of grain boundary corrosion [22]. With an addition of 2.0 wt % Mg to the Zn bath, a suitable amount of (Zn + MgZn_2_) eutectics resulted mainly in general corrosion [22]. With an addition of 3.0 wt % Mg to the Zn bath, the coarse and mesh-like grains give rise to severe localized corrosion [22]. It seems that the suitable content of (Zn + MgZn_2_) eutectics and morphology of MgZn_2_ are very important factors to improve the corrosion resistance of Zn-Mg coatings.

It was reported that when the content of Mg in the Zn-Mg alloy was less than 8.0 wt %, its corrosion resistance increased with the Mg content increasing [3]. However, according to the present results, it is found that the optimal corrosion resistance of the Zn-Mg coating containing 0.20 wt % Al can be obtained with an addition of 2.0 wt % Mg to the Zn bath. The reasons for this difference are associated with the phase composition and morphology of the microstructure. In the case of Zn-Mg alloys, Mg_2_Zn_11_ was found due to equilibrium solidification. However, in the case of Zn-Mg coatings, the transformation of MgZn_2_ to Mg_2_Zn_11_was inhibited due to the rapid solidification rate under the condition of hot-dip galvanizing [23]. Obviously, the phase composition difference will lead to a difference of corrosion resistance. As we know, the morphology of a phase is an important factor to determine the corrosion resistance [29]. The morphologies of Zn and Zn-Mg compound in the Zn-Mg coatings are different from that in the Zn-Mg alloys. Thus, this difference in morphology also results in a difference of corrosion resistance.

### 3.4. Characterization of Corrosion Products

**Figure 9:**
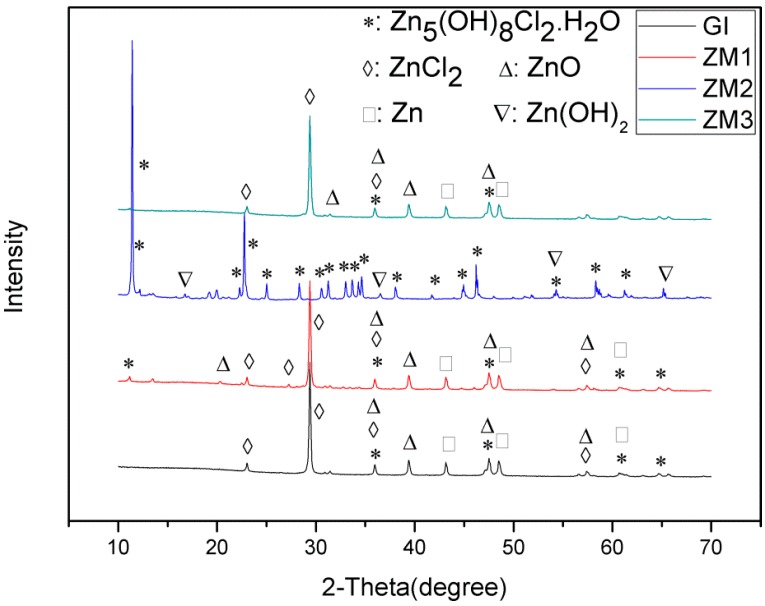
XRD patterns of corrosion products on GI and ZM after the salt spray experiment.

The morphology and phase composition of corrosion products are important factors to improve the corrosion resistance of Zn based coatings. Figure 9 shows XRD patterns of corrosion products on GI and ZM coatings after salt spray experiment. From Figure 9, it can be seen that corrosion products in crystal form consist of soluble ZnCl_2_, ZnO (zinc oxide), and Zn_5_(OH)_8_Cl_2_·H_2_O (simonkolleite) for GI, ZM1, and ZM3 coatings, respectively. In contrast, the corrosion products on the ZM2 coating consist of Zn_5_(OH)_8_Cl_2_·H_2_O, Zn(OH)_2_ without ZnCl_2_ and ZnO. Simonkolleite is the main species.

**Figure 10:**
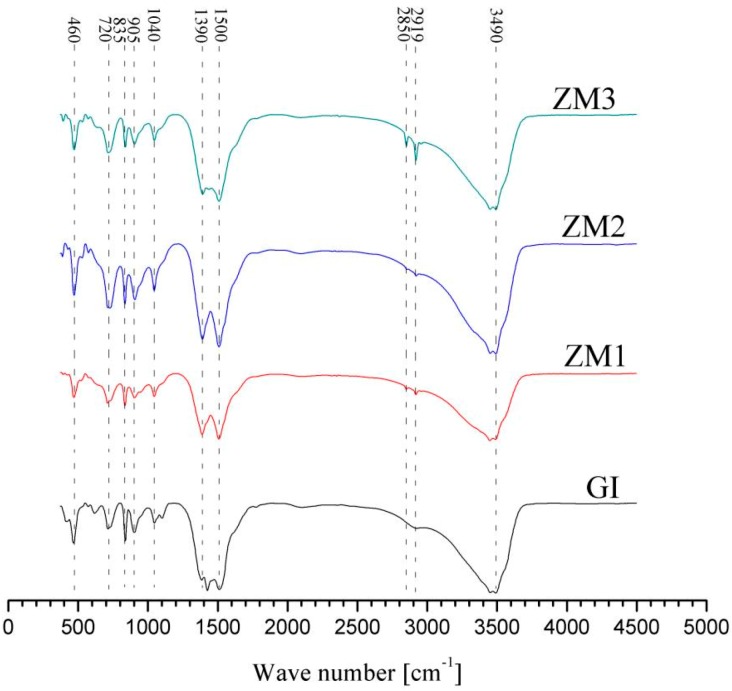
Infrared transmission spectra of corrosion products on GI and ZM coatings after the salt spray experiment.

Figure 10 shows infrared transmission spectra of corrosion products on GI and ZM coatings after the salt spray experiment. As seen in Figure 10, peak sites of infrared transmission spectra of corrosion products are almost the same, implying the corrosion products have identical phase composition. According to the previous study [3], the corrosion products can be identified easily. Broad peaks with the maxima at 3500–3400 cm^−1^ correspond to vibrations and rotations of water molecules and the OH^−1^ group. The peaks at 1500, 1390, and 835 cm^−1^ are ascribed to Zn_5_(OH)_6_(CO_3_)_2_ (hydrozincite). The peaks at 905 cm^−1^ and 720 cm^−1^ confirm the presence of simonkolleite in the corrosion products. The results of infrared transmission spectra analyses show the corrosion products on the GI and ZM coatings consist of hydrozincite and simonkolleite. The corrosion products on the GI and ZM coatings after the salt spray experiment can be identified combined with the results of XRD. The corrosion products of GI, ZM1, and ZM3 coatings consist of ZnCl_2_, ZnO, Zn_5_(OH)_8_Cl_2_·H_2_O, and Zn_5_(OH)_6_(CO_3_)_2_. The corrosion products of the ZM2 coating consist of Zn_5_(OH)_8_Cl_2_·H_2_O, Zn(OH)_2_, and Zn_5_(OH)_6_(CO_3_)_2_. Zn_5_(OH)_6_(CO_3_)_2_ and Zn_5_(OH)_6_(CO_3_)_2_ are believed to be a dense corrosion product with respect to porous ZnO, which can enhance the corrosion resistance of Zn coatings [30]. Zn(OH)_2_ is also supposed to be a beneficial corrosion product in improvement of corrosion resistance for Zn coatings [16]. Therefore, the enhanced corrosion resistance of ZM2 coating is related to its corrosion products based on the results of salt spray experiment. However, it seems that enhanced corrosion resistance of ZM1 and ZM3 coating cannot be ascribed to the difference in the phase composition of the corrosion products.

**Figure 11:**
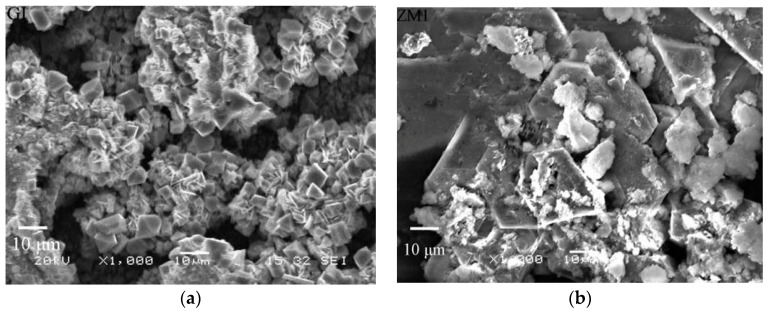

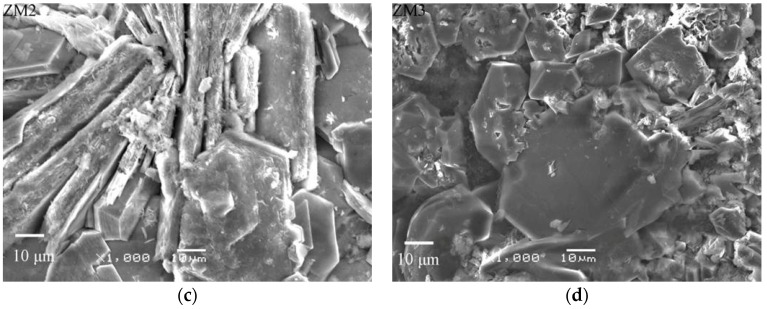
SEM micrographs of corrosion products on GI and ZM coatings after salt spray experiment, (**a**) GI; (**b**) ZM1; (**c**) ZM2; and (**d**) ZM3.

The corrosion resistance of Zn coatings is closely related to the morphology of the corrosion products. Figure 11 represents the corrosion morphologies of the corrosion products on GI and ZM coatings after the salt spray experiment. As seen in Figure 11, a large number of particle-like porous corrosion products and a large number of large holes on the corrosion surface of the GI coating are observed (Figure 11a).

**Figure 12:**
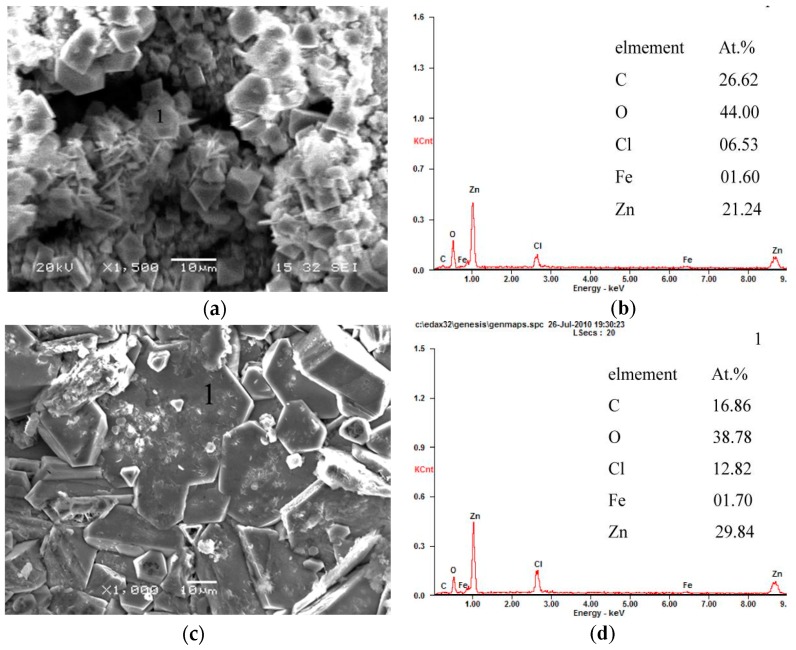
EDS analyses of representative corrosion products on GI and ZM3 coatings after the salt spray experiment. (**a**) GI; (**b**) EDS results of GI; (**c**) ZM3; and (**d**) EDS results of ZM3.

EDS analysis shows that these particles with a low Cl^−^ content (6.53 at %) may be ZnO (Figure 12a,b). These defects remarkably reduce the protection ability of the corrosion product layer to the steel substrate. For this reason, the corrosion resistance of GI coating is very inferior. In contrast, a large quantity of the sheet-like corrosion products on ZM coatings are observed (Figure 11b–d). EDS analysis shows these sheet-like corrosion products are probably simonkolleite (Figure 12c,d). Stoichiometry and XRD analysis will be conducted to test the corrosion products. Additionally, no corrosion holes are observed on ZM coatings. In this sense, enhanced corrosion resistance of ZM coatings is partly attributed to the dense morphology of corrosion products compared with GI coating. Among ZM coatings, a few particle-like ZnO particles are still observed for ZM1 and ZM3 coatings. However, no ZnO particles are observed for the ZM2 coating. It can be concluded that the best corrosion resistance can be acquired with 2.0 wt % Mg in ZM coatings in terms of the morphology of the corrosion products.

## 4. Conclusions

(1) Phase composition of the ZM coating containing 0.20 wt % Al is the same as that of Zn-Mg coating based on the vertical section of the calculated Zn-0.20 wt % Al-Mg ternary phase diagram near the Al-low corner.
(2) Morphology of Zn in the coating changes from bulk to strip, and finally to mesh-like, and MgZn_2_ changes from rod-like to mesh-like with Mg in the coating increasing from 1.0 to 3.0 wt %. The Mg added to the Zn bath can affect the formation of the Fe_2_Al_5_ inhibition layer.
(3) Compared to the GI coating, the corrosion resistance of ZM coatings improves by more than two-fold in terms of the time to first red rust. The expansion rate of red rust for ZM2 coating reduces by more than four-fold. ZM2 coating is the best corrosion resistant coating.
(4) The enhanced corrosion resistance of ZM1 and ZM3 coatings is related to the morphology of corrosion products and that of ZM2 is associated with not only the phase composition, but also the morphology of corrosion products.

## Figures and Tables

**Table 1:** Chemical composition of IF steel sheet (wt %).

C	Si	Mn	P	S	N	Al	Cr	Ni
0.009	0.008	0.091	0.0095	0.0093	0.0017	0.037	0.015	0.017

**Table 2:** Corrosion resistance of GI and ZM coatings evaluated by the salt spray test in 5 wt % NaCl solution.

Designation	Time to First White Rust (Day)	Time to First Red Rust (Day)	Time to Severe Red Rust (Day)	Area of Severe Red Rust (%)	Time to Corrosion Expansion (Day)
GI	2	8	18	16.8	10
ZM1	2	18	59	16.6	40
ZM2	2	18	59	15.4	40
ZM3	2	18	59	17	40
